# Biomimetic bone-like regeneration potentiality and strength development of Mg-Zn-Ca alloys for maxillofacial application

**DOI:** 10.1186/s12903-026-08429-w

**Published:** 2026-05-06

**Authors:** Heba A. Shalaby, Rehab Salah, Madiha A. Shoeib, Ghada A. Koronfoly, Marwa Hassan Mostafa, Hatem M. Ibrahim

**Affiliations:** 1 Dental Biomaterial, Faculty of Oral and Dental Medicine, Nahda University, Beni-Suef, Egypt; 2 Dental biomaterial, Faculty of Dentistry, Al Fayoum University, Al Fayoum, Egypt; 3 Department of Dental Biomaterial, Faculty of Oral and Dental Medicine, Nahda University, Beni-Suef, Egypt; 4 Central Metallurgical Research and Development Institute (CMRDI), Former head of surface treatment and corrosion department, Cairo, Egypt; 5 Dental Biomaterial, Faculty of Faculty of Dentistry, Al Fayoum University, Al Fayoum, Egypt; 6 Fixed and Removable Prosthodontics Departments, Oral and Dental Research Institute, National Research Centre, Cairo, Egypt; 7 Removable prosthodontic department, Faculty of Dentistry, King Salman International University, El-Tur, South Sinai Egypt; 8 Dental Biomaterials, College of Oral and Dental Medicine and Surgery, Misr University for Science and Technology (MUST), Giza, Egypt

**Keywords:** Mg1Zn0.6Ca, Mg6Zn0.6Ca, Electrochemical corrosion, Surface roughness, SEM with EDX, XRD, FTIR. Simulating body fluid, Biodegradable Mg-based alloy, Flexural strength, Bioactivity

## Abstract

**Background:**

This study evaluated the potential for biomimetic new bone regeneration synchronized with developing strength when the Zn proportion was increased in Mg-Zn-Ca alloys (Mg1Zn0.6Ca and Mg6Zn0.6Ca alloys).

**Methods:**

Two types of magnesium (Mg) alloys, Mg1Zn0.6Ca and Mg6Zn0.6Ca, with a total of 100 samples (*n* = 50/group), were studied. The groups were divided into subgroups (*n* = 10/group) A; subjected to electrochemical corrosion, (B, C, and D) subgroups were biomimetic immersed in SBF for different time intervals (2,4,and 8weeks), receptively, to assess the degradation/regeneration rate. Another subgroup (*n* = 10/group) were used as control for initial flexural strength test. Changes in weight were recorded, and surface chemistry was analyzed through X-ray diffraction (XRD) and FTIR. Surface morphology changes were examined via environmental scanning electron microscopy (SEM) and EDXA. The formation of new bone was estimated by assessing mineral content, crystallinity, hydroxyapatite (HA) maturity, matrix amount, and the calcium/phosphorus (Ca/P) ratio. Additionally, surface roughness (Ra) and flexural strength before and after the biomimetic immersion in SBF at different intervals were measured.

**Results:**

Mg1Zn0.6Ca (Group I) demonstrated controlled long-term degradation; weight loss corrosion rate reduced from 1.81 to 0.26 mm/year over 8 weeks, mature HA formation (65.5% crystallinity, 161% crystal maturity by 8 weeks), the Ca/P ratio increased from 0.29 ± 0.03 to 0.68 ± 0.03 and preserved mechanical competence (88.75 ± 0.03 MPa flexural strength). On the other hand, Mg6Zn0.6Ca (Group II) showed rapid initial passivation (Ra ∼4.87 μm at 2 weeks) but unsustainable performance because of premature embrittlement (50.87 ± 0.03 MPa), immature HA (44% maturity, 37.7% crystallinity decline), the Ca/P ratio dropped from 1.42 ± 0.03 to 0.35 ± 0.03 and persistent high degradation (~ 2 mm/year). Electrochemical corrosion revealed that group II showed a higher corrosion potential (Ecorr), Icorr, and corrosion rate value than group I. Group II (Mg6Zn0.6Ca) showed a higher biodegradation rate (about 7.37 ± 0.02 mm/year) and higher surface roughness than group I (Mg1Zn0.6Ca) over time.

**Conclusions:**

Mg1Zn0.6Ca (Group I) shows superior biomimetic synchronization for maxillofacial bone regeneration, achieving controlled long-term degradation, as it revealed a controlled degradation with stable hydroxyapatite formation that promotes osteointegration and sustained mechanical strength. It is recommended to be used in short and long-lasting load-bearing maxillofacial appliances. In contrast, Mg6Zn0.6Ca (Group II) degrades too rapidly, leading to premature strength loss and unstable mineralization. It is recommended in short loading- bearing maxillofacial appliance.1 wt% Zn is the ideal amount for biomimetic maxillofacial implants in accordance with ISO 10993-5 biomechanical thresholds.

## Background

Magnesium (Mg) alloys are increasingly utilized as biodegradable scaffolds for orthopedic and cardiovascular applications due to their lack of cytotoxic effects, impressive biodegradability, strong biocompatibility, and mechanical properties that are comparable to those of human bone [1].

However, the primary challenge limiting the widespread clinical application of pure Mg is its rapid degradation rate in the physiological environment. The aggressive corrosion of Mg in the presence of chloride ions leads to a premature loss of mechanical integrity and the excessive evolution of hydrogen gas, which can hinder the healing process [2].

The basic research on bioresorbable metals focuses on improving the mechanical properties of metals by designing alloys (compositional) and by metallurgical processes and controlling corrosion behavior by modifying the substrate or surface with coatings or other surface treatments [3].

Alloying magnesium with elements like zinc (Zn) and calcium (Ca) has shown great success in addressing this.The amounts of calcium and zinc added should not exceed 2% and 6% by weight, respectively, due to the corrosion tendency of the magnesium alloy. Mg-based alloys have limited medical uses due to their high rate of disintegration and fast production (due to degradation) of hydrogen gas bubbles, frequently in the first week after surgery [4].Magnesium is believed to be responsible for osteoconductivity as it can regulate osteoblast and osteoclast activities [5]. Zn is also regarded as a dietary component contained in the human body and consumed on a daily basis. It aids in cellular metabolism and has antimicrobial properties [6].

Recent advancements in magnesium-based alloys have shown its suitability for orthopedic and craniofacial implants, utilizing their density (1.7–2.0 g/cm³) that aligns with bone and a Young’s modulus (40–45 GPa) that diminishes stress shielding in comparison to Ti-6Al-4 V (110 GPa). Comprehensive assessments emphasize Mg-Zn-Ca systems as frontrunners because of adjustable degradation (0.2–1.0 mm/year), increased biocompatibility, and secondary phase strengthening [7]. Zn alloying (1–6 weight%) achieves ultimate tensile strengths of 240–340 MPa appropriate for temporary implants by refining grain size through Hall-Petch processes and preventing premature hydrogen development [8]. For load-bearing applications, surface bioactivation via the production of hydroxyapatite (HA) further synchronizes deterioration with new bone ingrowth [9].

Current titanium implants used in maxillofacial surgery have long-term side effects such as palpability (particularly in the orbital floor), peri-implantitis (15–20% occurrence), and the need for further surgeries to remove them. These constraints are addressed by biodegradable magnesium alloys, especially for orbital floor repair (30–70 MPa) and mandibular angle restoration (100–150 MPa flexural stresses), where 6–12-month resorption corresponds with bone healing timeframes [10]. The ideal Zn concentration, which ranges from 1 weight% (ductility focused) to 6 weight% (early passivation), is still debatable because it balances mechanical reinforcement, controlled corrosion, and biomimetic mineralization. Resolving these trade-offs requires systematic optimization using FTIR mineral/matrix profiling, flexural testing under masticatory simulation, and long-term SBF immersion.

Higher Zn content in the Mg-Zn system, on the other hand, is a neurotoxic [11]. Alloying with Zn has a number of good impacts on Mg alloys, including increased strength, grain refinement, enhanced castability, and corrosion reduction due to the formation of a passive layer on the alloy’s outer surface Zinc was added to magnesium alloys enhanced mechanical strength and enhanced the nucleation of hydroxyapatite that act as bone-like structure [12].

Calcium is a major constituent of hydroxyapatite Ca_5_(PO4)^3^(OH), the crystalline bone phase. Alloying Ca with Mg improved corrosion resistance when included in low quantities (0.6–0.8% being optimum It reduced the alloy’s grain size, improving the compressive and bending strength. Calcium is considered a nucleating agent for HA crystal growth. It can stimulate osteoblasts’ differentiation for mineral deposition [13].

This study investigated the biodegradable Mg-6 wt%Zn-0.6 wt% Ca alloy based on the Mg-Zn-Ca ternary system. Zinc (Zn) is considered an essential mineral for the human body, playing a critical role in the proper functioning of a variety of enzymes, as well as contributing to immune function, protein and DNA synthesis, and tissue repair. Due to their vital roles in human health, magnesium metal should be alloyed primarily with Zn and Ca for biomedical applications [14].

Monitoring the biodegradation behavior of Mg1Zn0.6Ca and Mg6Zn0.6Ca alloys to evaluate the biodegradation rate behavior synchronized with new bone-like mineral allowing bone regeneration in vivo, is very challenging.

The null hypothesis of this study is that biomimetic immersion of Mg6Zn0.6Ca alloy containing more percentage of zinc acts as a nucleating agent for HA crystals. Consequently, it might promote a higher amount of well- formed HA mineral rather than Mg1Zn0.6Ca.

## Methods

### Sample preparation and grouping

**Fig. 1:**
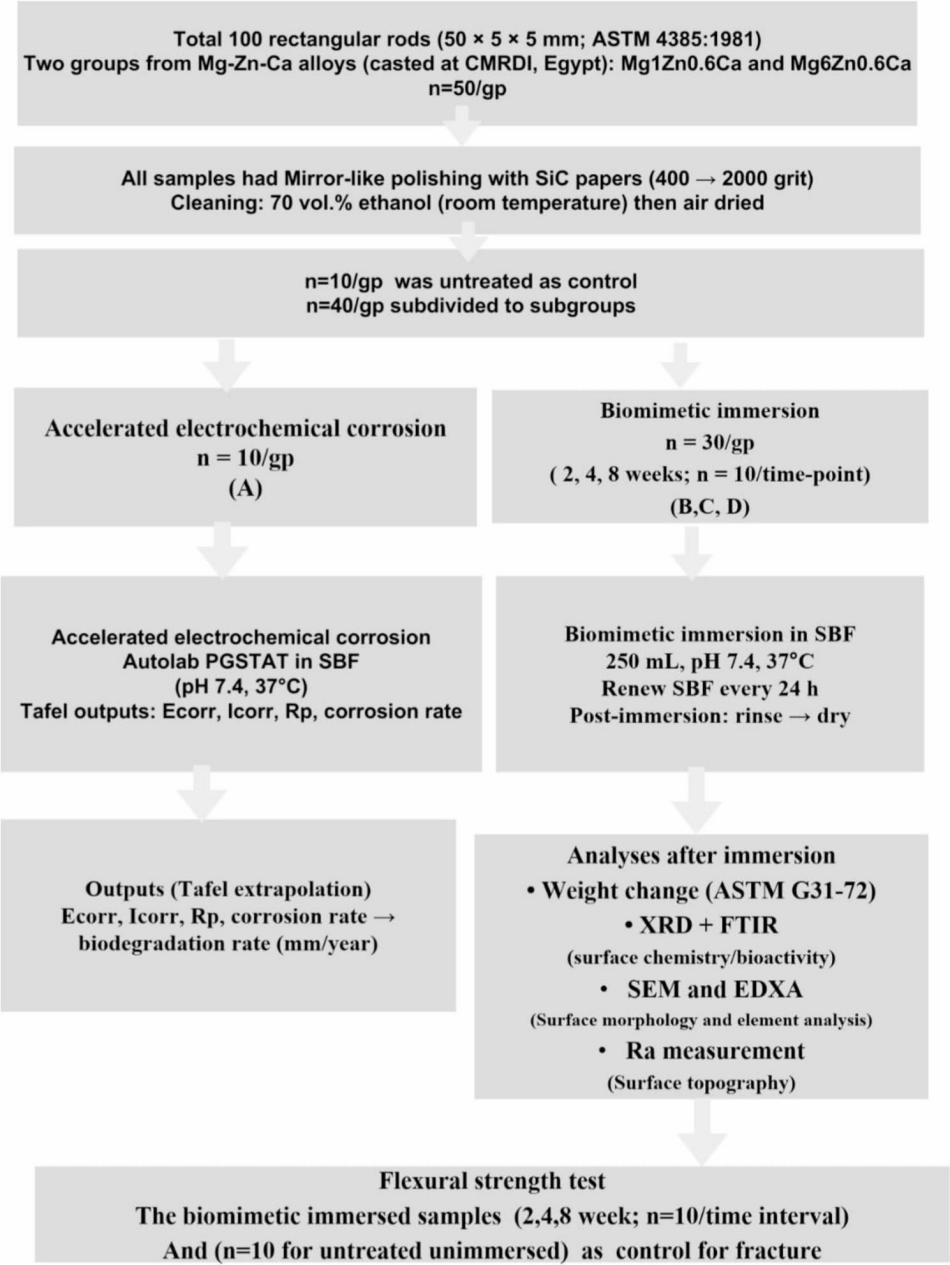
flowchart

A total of 100 rod samples of two different magnesium zinc calcium alloys were used in this study: Mg1Zn 0.6Ca and Mg6Zn0.6Ca alloy (casted in The Central Metallurgical Research and Development Institute (CMRDI), Egypt), compositional constituents of Mg alloys used were assessed by XRF, are shown in Table (1), Ibrahim H M et al.,2018 [15]. Group I was Mg1Zn 0.6Ca, and group II was Mg6Zn0.6Ca, (*n* = 50/group). The groups (*n* = 40) were divided into subgroups A, B, C, and D (*n* = 10), in which sub-group A was subjected to electrochemical corrosion test. Whereas, B, C and D sub-groups were biomimetic immersed in SBF for (2, 4, 8 weeks), respectively. The rest of samples of each group (*n* = 10) was subjected to the flexural strength test as a control group (reference before biomimetic degradation). The samples were prepared according to ASTM standard 4385:1981 as rectangular rods with dimensions of 50 × 5 × 5 mm; surface area10.5 cm². The sample’s surface was polished mirror-like using Emery SiC paper, with a grit blasted range from 400 to 2000 then cleaned with 70 vol% ethanol at room temperature and dried with an air drier [16]. Control group of both types of alloys was chemically characterized by using XRD and FTIR, surface roughness measured and morphology was studied by SEM and EDXA before subjected to electrochemical corrosion and Biomimetic immersion in SBF. Figures (1): Flowchart.

**Table 1:** Chemical composition of the tested magnesium alloys in wt%

Alloys	Chemical composition (wt%)				
	Zn	Ca	Fe	Cu	Mg
Mg1Zn0.6Ca	1.18	0.514	0.031	0.045	balance
Mg6Zn0.6Ca	6.34	0.59	0.032	0.036	balance

## Accelerated electrochemical corrosion test

The two types of tested alloys IA and IIA (*n* = 10/group) were subjected to the accelerated Electrochemical biodegradation tests by using Autolab Potentiostat/ Galvanostat (AUTO LAB PGSTAT 302 N, Switzerland) controlled by Nova software (Nova, 1.10.2, Metrohm Autolab B.V.) in SBF at pH 7.4 and 37 ± 0.5 °C to evaluate the accelerated biodegradation rate in mm^− 1^/year for all alloys, according to *Shalaby and Shoeib 2017* [17]. The tested groups were used as the working electrode, a platinum rod as the auxiliary electrode and a saturated calomel electrode as the reference electrode. The area of the working electrode exposed to the solution was 10 mm×10mm^2^. The potential was changed from cathode direction to anodic direction in the range − 0.4 to 1 V with scan rate 5 mV/sec. Corrosion current (ICorr) of each sample, corrosion potential (ECorr), potential resistance (Rp) and corrosion rate was estimated digitally from Tafel extrapolation curve.

## Biomimetic immersion degradation rate testing

### Biomimetic immersion of prepared samples

The tested samples were suspended in a properly sealed beaker filled with 250 ml SBF solution (Kokubo recipe, ISO 23317:2006; 20 mL/cm² ratio) at 37 ± 1 °C (orbital shaker, 100 rpm), initial pH 7.40 ± 0.05 (daily monitored, no active buffering), refreshed weekly, for 2, 4, and 8 weeks. SBF composition: 8.0 g/l NaCl, 0.14 g/l CaCl_2_, 0.4 g/l KCl, 0.35 g/l NaHCO_3_, 1.00 g/l C_6_H_12_O (Glucose), 0.1 g/l NaH_2_PO4, 0.1 g/l MgCL_2_.6H_2_O, 0.06 g/l Na_2_HPO_4_2H2O, 0.06 g/l MgSO_4_.7H_2_O). The samples were static immersed in SBF at 37 °C for 2, 4, and 8 weeks (*n* = 10/group), according to Kokubo et al. 2006 and Oyane et al. 2005) [18, 19]. At the end of each immersion period, the specimens were removed from the solution, rinsed with distilled water to remove any loose surface corrosive products (loose ions/salts only), while corrosion/Remineralization products (Mg(OH)_2_, HA) preserved and finally dried at room temperature, to quantify net mass changes reflecting biodegradation and biomineralization, according to Shalaby et al. 2016 and Rahim et al. 2020 [20, 21].

### Weight change measuring

The biodegradation rate was determined using the weight-loss/gain (degradation/mineralization) method. The biodegradation rates (mm/hr) were determined according to ASTM-G31-72 by the following equation:

$$\text{Degradation rate}=\left(\mathrm{K} \times \mathrm{W} \right) \div \left(\mathrm{A} \times \mathrm{T} \times \mathrm{D} \right)$$

Where K = a conversion constant (8.76 × 10^3^) for mm/year, W = mass loss in (mg), A = Exposed surface area (cm²; 10.5 cm² for 50 × 5 × 5 mm rods), T = time of exposure in hours to the nearest 0.01 h. and D = density in g/cm^3^ ( 1.80 g/cm³ for Group I, 1.85 g/cm³ for Group II) *Katarivas et al.*,* 2017 and* Rahim et al. 2020 [22, 21].

The surface chemistry identified (qualitative and quantitative) of tested alloys surface at different time intervals by XRD and FTIR. Surface morphology changes were examined via environmental scanning electron microscopy (SEM) and EDXA.

The electrochemical corrosion versus weight changes were estimated by calculating the % difference between corrosion rate potential and biodegradation rate (weight change) by using the following formula;[23, 24].

$$\begin{aligned} {\%}\,\mathrm{Difference}&=\left[\left(\text{CR-potentio- CR weight}\right) \right.\\&\left./ \mathrm{CR-weight}\right] \times\;100 \end{aligned}$$

Where, CR- potentio is potentiodynamic corrosion rate (mm/year), CR- weight is weight loss corrosion rates (mm/year).

### Qualitative chemical analysis

A thin film X-ray diffractometer with a copper target (Cu kα = 1.54060) and nickel filter (PAN analytical, X’Pert Pro, Holand) was used. The mathematical procedures were facilitated by computer software (PSI-Plot, poly software international, Salt Lake City, Utah, USA). The XRD data were based on Bragg’s equation *Bragg and Bragg. 1913* [25].

$$\mathrm{n}\lambda=2d\;\sin\theta$$

Where n = Integral number, λ = Wavelength, d = Interplanar space and θ = Diffraction angle.

Fourier transforms infra-red spectra (FTIR) (VERTEX70 model, Germany)) from 400 to 4000 cm^− 1^ of the different alloys’ groups were obtained by reflection at different time intervals 0, 2, 4 and 8 weeks. Qualitative analysis was employed by interpreting the corresponding functional groups to the specific bands’ wave numbers.

### Quantitative bioactivity chemical analysis

Biomimetic bioactivity of different tested groups was evaluated by calculating the quantitative amount of different chemical crystalline and organic compounds formed on the tested alloys surface to detect the amount of minerals and matrix after immersed in SBF that represented newly formed like-bone and compared to natural bone as follows.

The peak area changes of XRD graph of different chemical phases were calculated at different time intervals (0, 2, 4, and 8 weeks) by using software for analysis (PSI-Plot, Poly Soft Ware International, Salt Lake City, Utah, USA), according to according to *Rehab et al.*,* 2023* [26].

Quantitative analysis of FTIR of different tested groups was employed by calculating the full width at half maximum (FWHM) of reflected spectra, according to methodology described by *Cavalli et al.*,* 2014* [27]. Calculating Mineral quality by phosphate contents (PO4), degree of HA crystal maturity (CO3/PO4) and degree of crystallinity. Whereas matrix was evaluated by calculating the amount of amide I and the degree of calcification (mineral to matrix ratio) was evaluated from (PO4/Amide I), as shown in Table (2), according to *Rehab et al. .*,*2023* [26]. The data of the deposited layer was compared with actual human bone according to *Farlay D.*,* 2010* [28].

**Table 2:** Shows the selected parameters to assess bone quality

Parameter		IR peaks assignment	Comments
Mineral Quality	PO_4_^-3^	Integrated area of v_1_, v_3_ (900-1200 cm^-1^) phosphate	Mineral content
	CO_3_/ PO_4_	Integrated area of sub-band at 873 cm^-1^/ 1030 cm^-1^	Degree of HA crystal maturity or indicates the amount of carbonate substitution for phosphate in the mineral crystals
	Crystallinity Index or Splitting Factor	Summing the heights of the 563 and 603 cm^-1^ peak and dividing this value by the height of the trough between them	Degree of HA crystallinity
Matrix Quality	Amide I	Amide I (1575-1720 cm^-1^)	Protein contents in matrix
Mineral/Matrix Ratio	PO_4_^-3^/Amide I	Integrated area of v_1_, v_3_ (900-1200 cm^-1^) phosphate/amide I (1575-1720 cm^-1^)^101^	Degree of calcification

### Surface topography and morphology

The tested Mg alloy samples before and after biodegradation were gold sputtered using Hummer 5 sputter coater. Each group’s surface morphology was examined using a scanning electron microscope (Zeiss sigma 500vp analytical FE-SEM Field Emission scanning electron microscope) at different magnifications. Then the surface topography was monitored to record the average surface roughness (Ra). The amount of Ca, P, and Ca/P ratio deposited on the immersed alloys was detected by using EDX.

### Flexural strength

The intact samples of alloys and the biomimetic immersed alloys of different sub-groups B, C, and D (*n* = 10) were subjected to the three-point loading fracture test by using a universal Instron testing machine (USA, System ID:3366L8668). The indenter was loaded at a speed of 2 mm/min till the tested samples fractured. The flexural strength of different tested groups was calculated according to the following equation: R= ¼ 3FL/ 2bh2.

Where R is the flexural strength, F is the loading force, L is the span length, b is the width of the sample, and h is the height of the sample.

### Statistical analysis

Data was collected and analyzed with SPSS for windows version 20, and the findings revealed a normal distribution using the Shapiro Wilk test. One-way ANOVA was used to compare the groups, followed by a Tukey post hoc test. P-value was considered significant at ≤ 0.05.

## Results

### Accelerated electrochemical corrosion results

**Fig. 2:**
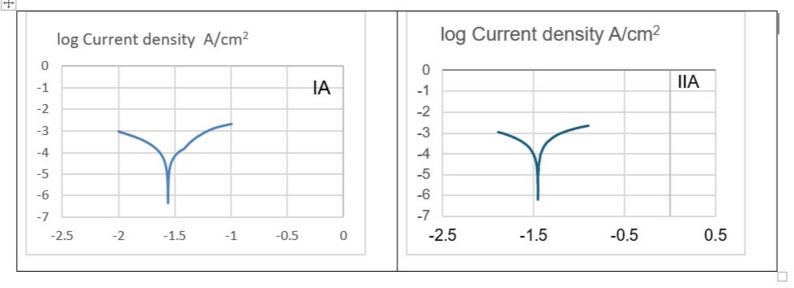
Potentiodynamic polarization curves (Tafel extrapolation) of group IA and group IIA

The electrochemical parameters results: current density (Icorr), Polarization resistance, and Corrosion potential (Ecorr) of the different tested groups of alloys that were subjected to accelerated electro chemical corrosion in SBF for 20 min are shown in Table (3) and Fig. (2). Tafel extrapolation curve of group I revealed negative shifting to the anodic part on contrary to group II that shifted toward the positive or Cathodic part, Fig. 2(A) Group I has Current Density (icorr), Polarization Resistance, Corrosion Potential (Ecorr), and Corrosion Rate ; 72.46 ± 0.95 µA/cm², 0.905 ± 0.059 KΩ, -1.751 ± 0.095 V, and 4.23 ± 0.05 mm/year, respectively. On other hand group II has ~ 72% significant higher current density, ~ 48% lower polarization resistance, less negative Ecorr and corrodes ~ 74% faster than Group I, as shown at Table (3) and Fig. 2(B).

**Table 3:** The statistical data of electrochemical corrosion parameters of tested groups in SBF (current density Icorr, Polarization resistance, Corrosion rate, and Corrosion potential Ecorr)

Groups.	Current density (icorr) (μA/cm2)	Corrosion rate (mm/year)	Polarization resistance(KΩ)	Corrosion potential (Ecorr) (V)
	Mean±SD	Mean±SD	Mean±SD	Mean±SD
Mg1Zn0.6Ca (gp I)	72.4577±0.9478	4.23±0.05	0.90517±0.059498	-1.751± 0.094569
Mg6Zn0.6Ca(gp II)	124.6725±0.6437	7.137±0.02	0.4717±0.069875	-1.663± 0.1035
*p*-value	p<0.05	<0.001	p<0.05	p<0.05

### Biomimetic immersion degradation rate results

#### Weight change measuring results

**Fig. 3:**
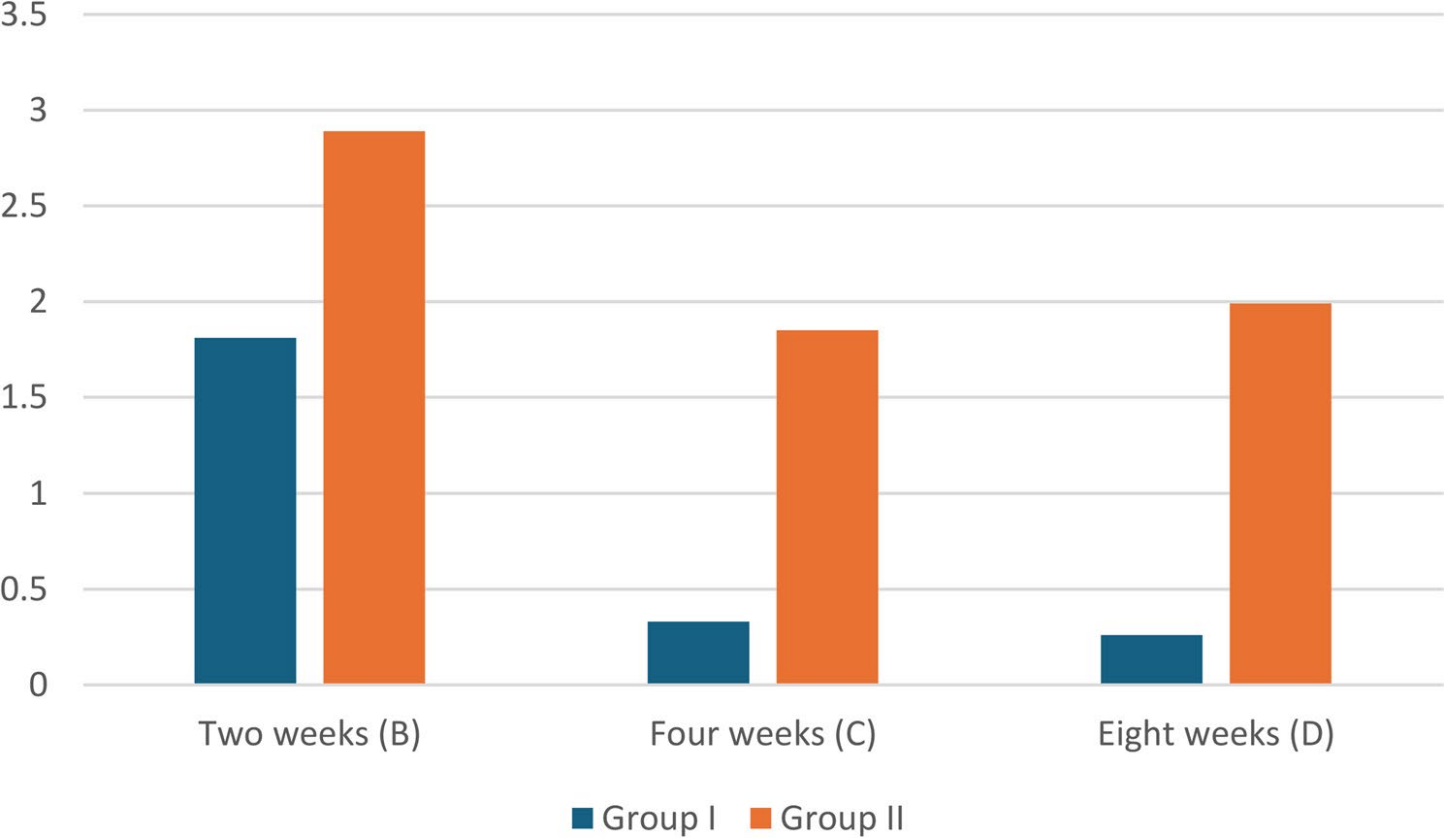
Histogram representing the degradation rate (mm/hr) of the tested magnesium alloys after biomimetic immersion in SBF at different time intervals

The statistical analysis for the degradation rate of biomimetically immersed alloys in SBF at different time intervals; 2, 4, and 8 weeks are presented in table (4) and fig. (3). Within Groups Comparison over time, Group I revealed degradation rate decreased sharply from 1.81 mm/year at two weeks to 0.33 and 0.26 mm/year at four and eight weeks, respectively. Group II revealed decreased degradation rate from 2.89 mm/year at two weeks to around 1.85-1.99 mm/year at four and eight weeks. Group II consistently exhibited a significantly higher degradation rate than Group I at all time intervals.

**Table 4:** Descriptive statistics and test of significance for degradation rate (mm/year) of the tested magnesium alloys after biomimetic immersion in SBF at different time intervals (2, 4, and 8 weeks)

Groups	Group I	Group II	P-value for each row
	Degradation rate (mm /year)		
Immersion time	Mean ± SD	Mean ± SD	
Two weeks (B)	1.81 ± 0.43^Ab^	2.89±0.39^Aa^	<0.001
Four weeks (C)	0.33 ± 1.6^Bb^	1.85±0.4Ba	<0.001
Eight weeks (D)	0.26 ± 0.35^Bb^	1.99±0.44^Ba^	<0.001
P-value for each column	<0.001	<0.001	

Friedman test for comparison along time interval P-Values for degradation rate along the same group; represents the significance along the same column by upper letter (time intervals). Small letter represents the significance difference along rows between groups for each time interval

*SD* Standard deviation, *P* Probability level

##### The electrochemical corrosion rate versus biodegradation weight changes

The % difference comparison between potentiodynamic corrosion rate and weight changes revealed massive divergence in group I; potentiodynamic severely overestimates (134-1527%) and the passivation occurred weight loss drops dramatically (1.81 → 0.26 mm/year). Group II potentiodynamic moderately overestimates (147-258%) and limited protection, Weight loss stabilizes ~2 mm/year vs initial 2.89, table (5). 

**Table 5:** The %difference between potentiodynamic corrosion rate and weight changes after immersion in SBF at different time intervals 2/4/8 weeks

Time point	Group I: Weight Loss	Group I: Potentiodynamic	Group II: Weight Loss	Group II: Potentiodynamic
2 weeks	1.81 mm/year	4.23 (↑134%)	2.89 mm/year	7.137 (↑147%)
4 weeks	0.33 mm/year	4.23 (↑1182%)	1.85 mm/year	7.137 (↑286%)
8 weeks	0.26 mm/year	4.23 (↑1527%)	1.99 mm/year	7.137 (↑258%)

#### Qualitative and quantitative chemical analysis

All surfaces of the tested groups were analyzed qualitatively and quantitatively before and after the biomimetic degradation at different time intervals.

##### Qualitative XRD analysis results

**Fig. 4:**
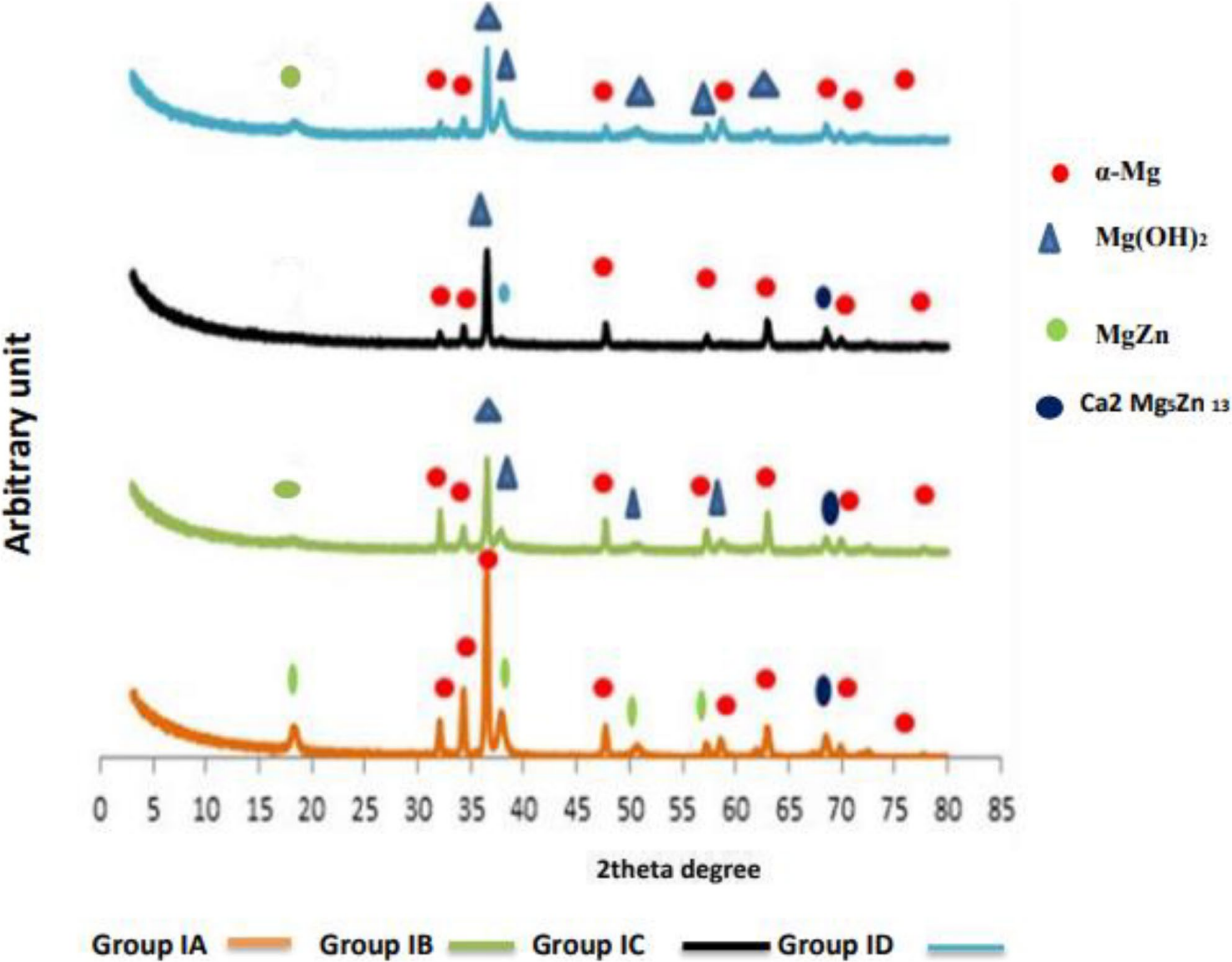
The diffractographs represent different chemical phases of group IA, IB, IC and group ID

The chemical constituents’ phases of different tested groups were identified by XRD and calculated as shown at Table (6) and Fig. (4). Group I showed primary magnesium phase (α-Mg) also the intermetallic phase (Mg Zn), and Ternary intermetallic phase (Ca₂Mg₅Zn₁₃). The Mg (OH)₂ compound formed after two weeks from immersion. Trace amount of Hydroxyapatite (HA, bioactive phase) was detected in group I after 4 weeks only.

**Table 6:** Statistical analysis results of different chemical phases’ surface area changes of group I and II at different time intervals (2, 4, 8 weeks) before and after biomimetic immersion in SBF

Surface area of different phases (µm)									
Groups	Group I Mg1Zn0.6Ca				Group II Mg6Zn0.6Ca				*P*-value
time intervals (weeks)	A(0)	B(2w)	C(4w)	D(8w)	A(0)	B(2w)	C(4w)	D(8w)	
Phases									
α-Mg	3791.4± 0.01(83.03 %)	4863.8 ± 0.06(30.45 %)	10519 ± 0.22(99.9%)	445.6774 ± 0.03 (2.95%)	334.5±0.003(97.6 %)	10284 ±0.02(58.8%)	288±0.04 (15.3 %)	507 ± 0.03(10.9 %)	<0.001
Mg Zn	773±0.20(16.93 %)	0 ± 0	0 ± 0	0 ± 0	4.7 ± (1.37 %)	0 ± 0	0 ± 0	0 ± 0	<0.001
Mg(OH)_2_	0 ± 0	11102 ± 0.02(69.5%)	0 ± 0	15069.71±0.002(97.0%)	0 ± 0	1515 ± 0.002(8.7 %)	1598 ± 0.002(84.7 %)	4140 ± 0.01 (89.1%)	<0.001
Ca_2_Mg_5_Zn_13_	2.3 ± 0.12 (0.05 %)	4.48 ± 0.25(0.02 %)	3.27 ± 0.17(0.03 %)	0 ± 0	3.54(1.03%)	5686 ±(32.5%)	0 ± 0	0 ± 0	<0.001
Hydroxy-apatite(HA)	0 ± 0	0 ± 0	6.88 ± 0.29 (0.065%)	0 ± 0					<0.001

**Fig. 5:**
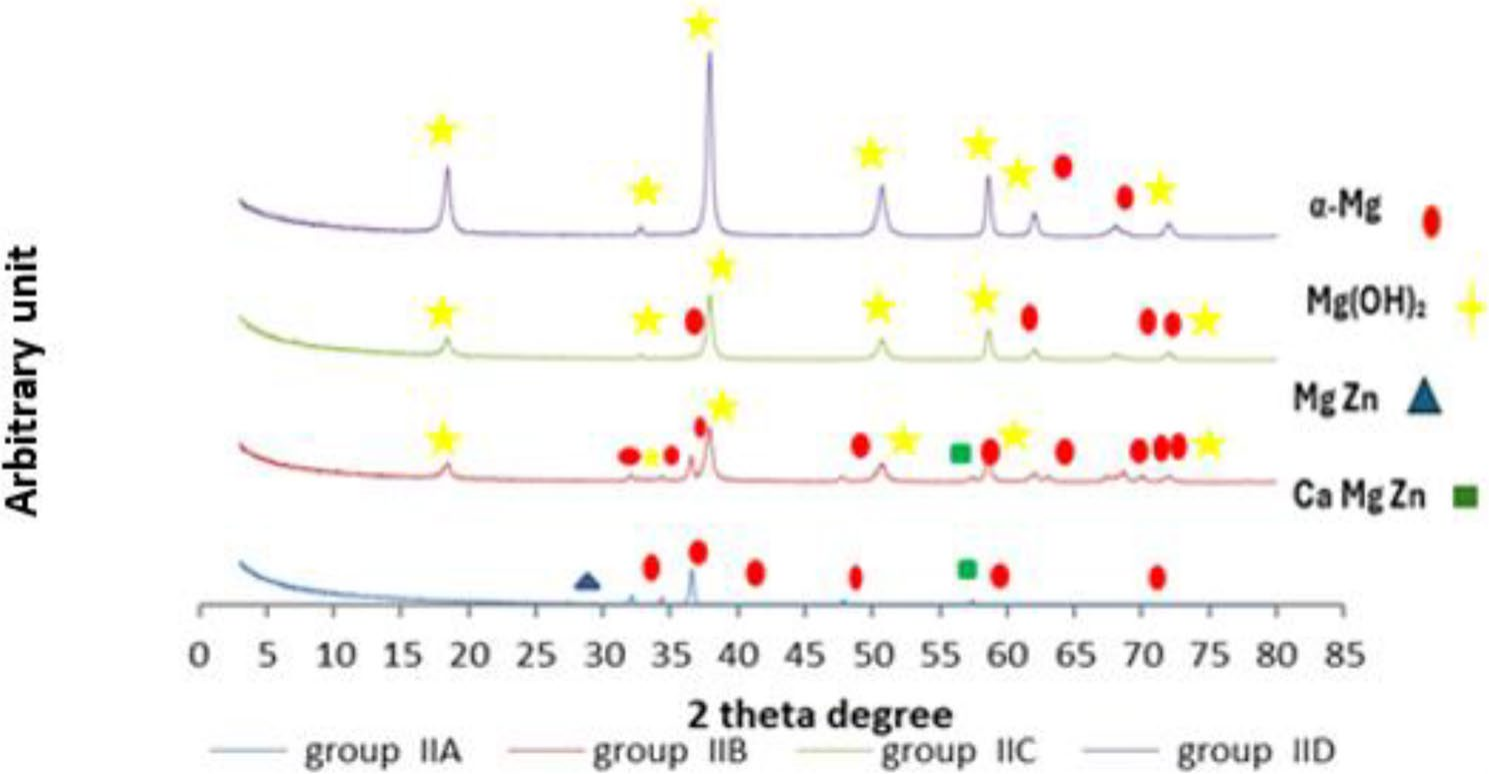
The diffractographs represent different chemical phases of group IIA, IIB, IIC and group IID

While the chemical constituents’ phases of different tested groups were identified by XRD and calculated as shown at Table (6) and Fig. (5). group II showed primary magnesium phase (α-Mg) also the intermetallic phase (Mg Zn), and Ternary intermetallic phase (Ca₂Mg₅Zn₁₃). the Mg(OH)₂ compound formed after two weeks from immersion.

##### Quantitative XRD analysis results

The chemical constituents’ phases of different tested groups were identified by XRD and calculated as shown at Table (6). Both groups showed primary magnesium phase(α-Mg); in group I; Started as the dominant phase (83%), sharply increased at 4 weeks (almost 100%), then dropped at 8 weeks (to 2.95%). In group II, it also started high (58.8%), but rapidly decreased over time, reaching 10.9% at 8 weeks. The intermetallic phase (MgZn) Present only at baseline (16.93%) in group I and (1.37%) in group II then disappeared after immersion. Mg (OH)₂ compounds formed after two weeks from immersion in both types of alloys; in group I 69.5% that disappeared after 4 weeks then dominated at 8 weeks (97%). Whereas in group II was (8.7%), then increased to dominate at 8 weeks (89.1%). Trace amounts of Ternary intermetallic phase (Ca₂Mg₅Zn₁₃) was detected at the base line until 4 weeks of both types of alloys that surged at 2 weeks in group II (32.5%) and disappeared at 8 weeks for both groups. Trace amount of Hydroxyapatite (HA, bioactive phase) was detected in group I after 4 weeks only.

##### The FTIR analysis results of group I and II

**Fig. 6:**
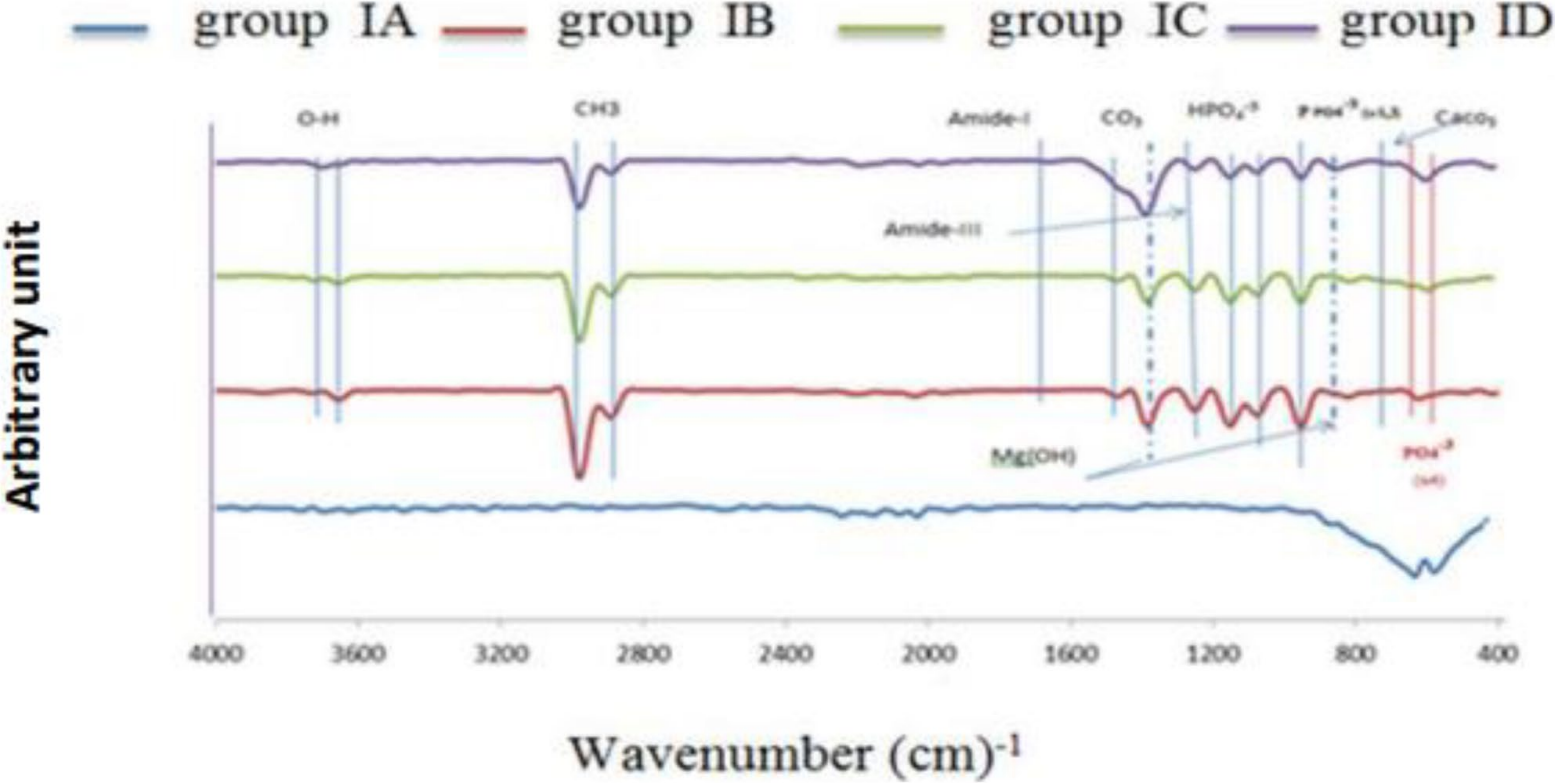
FTIR spectra represents different chemical compositions of group IA, IB, IC and ID

**Fig. 7:**
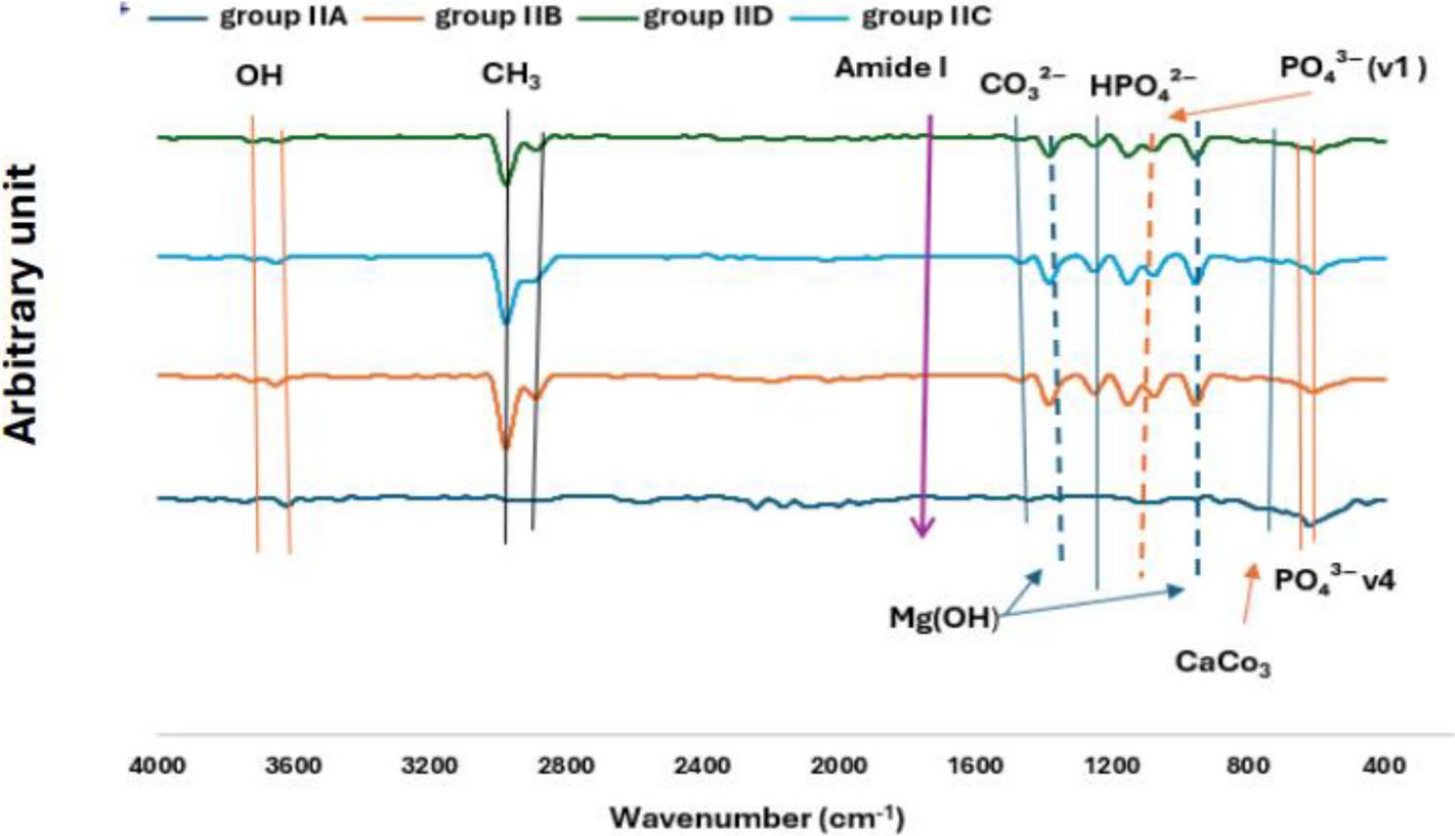
FTIR spectra represents different chemical compositions of group IIA, IIB, IIC and IID

The FTIR spectra analysis for both groups I and II showed the presence of Mg–O, CaCO₃, and Zn–O bands initially. After immersion in simulated body fluid (SBF). Both groups exhibited new peaks corresponding to PO₄³⁻ (ν₄ and ν₁), HPO₄²⁻, and CO₃²⁻, indicating successful hydroxyapatite (HA) deposition. Both groups demonstrated precipitation of Mg(OH)₂. Adding to that the functional groups such as amide I, CH₃, and OH were detected in both groups, reflecting the organic matrix components and surface chemistry, as shown in Figs. (6, 7).

The FTIR spectral changes after SBF immersion were similar in both groups, suggesting comparable biomimetic mineralization behavior. The presence of HA-related phosphate and carbonate groups in both groups confirms effective HA formation on the alloy surfaces.

The quantitative analysis of FTIR spectra revealed that amount of mineral content (PO₄) remained critically lower than the normal value (120.79 ± 0.45) in both types of alloy groups with minimal improvement over time in group I (0.0025–0.014% of normal) whereas group II showed no significant progression along the time intervals (0.016–0.02% of normal). The FTIR detection of phosphate groups confirmed that HA formation is initiated, but the very low quantitative mineral content suggested this deposition is minimal or poorly integrated.

HA Crystal Maturity (CO₃/PO₄) in normal bone is (1.54 ± 0.48). HA Crystal Maturity of group I started at 77.9% normal, declined to 53.9% at 4 weeks, then increased to 161% by 8 weeks. Whereas group II dropped to 24% of normal initially, and further declined to 20% in 4 weeks, with partial recovery to 44% by 8 weeks. HA Crystallinity in normal bone is 24.9 ± 0.18. The degree of HA crystallinity in group I Improved from 20.6% to 65.5% of normal by 8 weeks.Whereas group II reached 57.4% at 4 weeks but declined to 37.7% by 8 weeks.

The matrix quality (Amide I) is 10.28 ± 0.001 in normal bone. Both Groups revealed collagen matrix integrity is nearly absent (0.008% of normal) at all time intervals. The mineral/matrix ratio (PO₄⁻³/Amide) is 11.75 ± 0.002 in normal bone. The mineral/matrix ratio of group I fluctuated widely (28.9% to 216.5% of normal), peaking at 4 weeks. While group II was consistently subnormal (17–25% of normal).

#### Surface topography

**Fig. 8:**
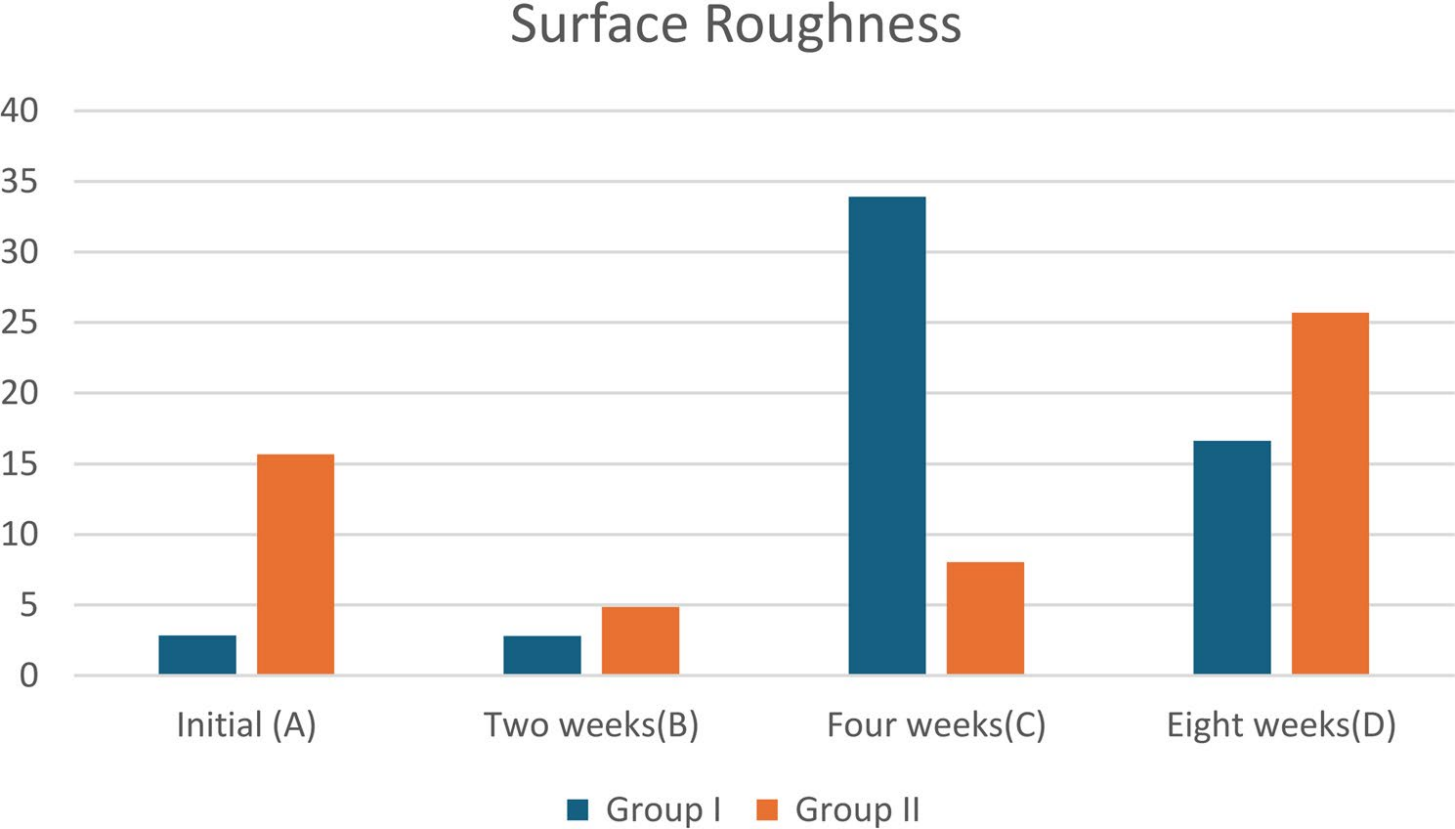
Surface roughness [µm] of the tested groups

The statistical analysis results for the Initial Surface Roughness surface roughness of Group I (Mg1Zn0.6Ca) revealed very low roughness (2.84 ± 0.03 μm), indicating a smooth initial surface. On the contrary, Group II (Mg6Zn0.6Ca) showed much higher initial roughness (15.69 ± 0.03 μm), suggesting a rougher starting surface. Both groups showed highly significant changes in surface roughness over time (*p* < 0.001) after immersion in SBF. Roughness of the group remains almost unchanged after 2 weeks (2.81 ± 0.03 μm) then sharp increase at 4 weeks (33.92 ± 0.03 μm), indicating significant surface changes, likely due to corrosion or biomineral deposition. Finally, after 8 weeks, roughness decreases to 16.64 ± 0.03 μm but still higher than initial values. In group II (Mg6Zn0.6Ca), the roughness decreased significantly from initial 15.69 ± 0.03 μm to 4.87 ± 0.20 μm in 2 weeks. Then gradually increased at 4 weeks (8.05 ± 0.18 μm) and further increased at 8 weeks (25.69 ± 0.5 μm), as shown in Table (7) and Fig. (8).

**Table 7:** Statistical analysis results of surface roughness [µm] for groups I and II before and after biomimetic immersion in SBF

surface roughness [µm]	Group I (Mg1zn0.6ca) Mean ± SD	Group II(Mg6zn0.6ca)Mean ± SD	*P*-value for each row
Initial (A)	2.84 ± 0.03^C^	15.69±0.03^B^	<0.001
Two weeks(B)	2.81 ± 0.03^C^	4.87±0.20^D^	<0.001
Four weeks(C)	33.92 ± 0.03^A^	8.05±0.18^C^	<0.001
Eight weeks(D)	16.64 ± 0.03^B^	25.69±0.5^A^	<0.001
*P*-value for each column	<0.001	<0.001	<0.001

Friedman test for comparison along the time interval *p*-value for roughness along the same group represents the significance along the same column by upper letter. The values with the same letter are non-significant. Whereas the different letter are significant

*SD*standard deviation, *p*probability level

#### Surface morphology

**Fig. 9:**
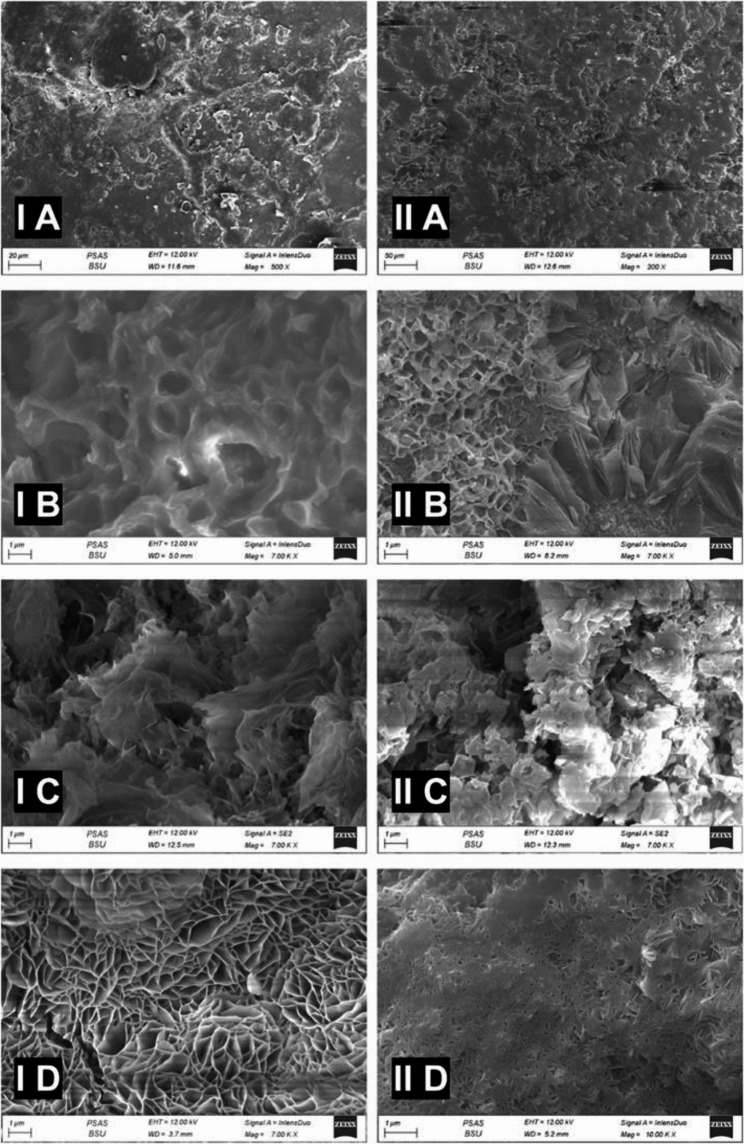
SEM image for both alloy before and after immersion

The main microstructural constituents of group IA (Mg1Zn 0.6Ca alloy) are shown in the SEM micrograph (Fig. 9; IA, IB, IC, ID). The dark gray areas refer to the matrix of the alloys α-Mg, while the large opaque globule is the MgZn phase. The small opaque globule was Ca_2_Mg_5_Zn_13_ phase, as illustrated in Fig. (9 IA). After two weeks (group IB); Mg (OH)_2_ phase (Brucite) appeared as an opaque phase covering a darker matrix of the alloys α-Mg, while the small opaque globule was identified as a Ca_2_Mg_5_Zn_13_ phase, Fig. (9 IB). The surface changed after four weeks to be opaque foggy or clouds shape that refers to amorphous calcium phosphate, Fig. (9 IC). Finally became hay-like after eight weeks, Fig. (9 ID).

The main microstructural constituents of group II (Mg 6Zn 0.6Ca alloy) are shown in the SEM micrograph Fig. (9: IIA, IIB, IIC, IID). When the alloy was immersed in SBF, the dark gray areas from the matrix of the alloys α-Mg was observed, large opaque globule was the MgZn phase with small scattered opaque globule for Ca_2_Mg_5_Zn_13_ phase distributed, as in Fig. (9 IIA). After two weeks, the opaque phase, Mg(OH)_2_ phase (Brucite) covered the matrix, Fig. (9: IIB) that changed to large plates of amorphous calcium phosphate after four weeks (group IIC) .

#### The Ca/P ratio

**Fig. 10:**
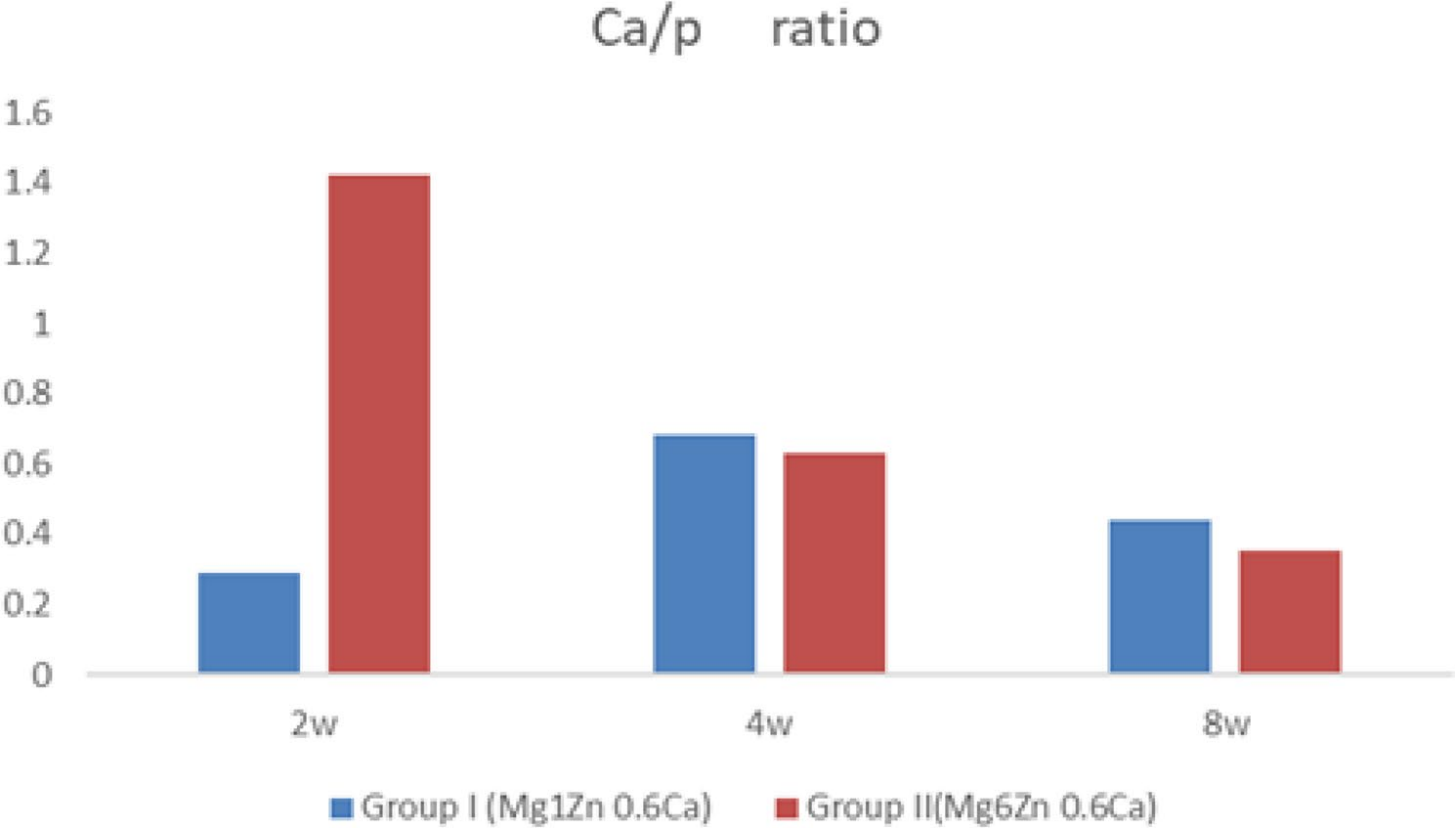
The Ca/P ratio for groups I and II after biomimetic immersion in SBF

The Ca/P ratio of calcium phosphate compounds formed on Mg alloys’ surfaces was estimated from EDX analysis after immersion at different time intervals (2, 4, and 8 weeks), shown in Table (8) and Fig. (10). In two weeks, Group II revealed a significantly higher Ca/P ratio than Group I (1.42 ± 0.03And 0.29 ± 0.03), respectively. After four and eight weeks, the ratios were closer, but Group I was slightly higher than group II (0.68 ± 0.03vs. 0.63 ± 0.03, *p* = 0.009), (0.44 vs. 0.35, *p* < 0.001), respectively.

**Table 8:** Mean and standard deviation (SD) of the Ca/P ratio for groups I, and II after biomimetic immersion in SBF

Groups	Time interval			*P*-value for each row
	Two weeks (B)	Four weeks(C)	Eight weeks(D)	
Group I (Mg1Zn 0.6Ca)	0.29 ± 0.03	0.68 ± 0.03	0.44 ± 0.03	<0.001
Group II(Mg6Zn 0.6Ca)	1.42±0.03	0.63±0.03	0.35±0.03	<0.001
*P*-value for each column	<0.001	0.009	<0.001	<0.001

#### Flexural strength

**Fig. 11:**
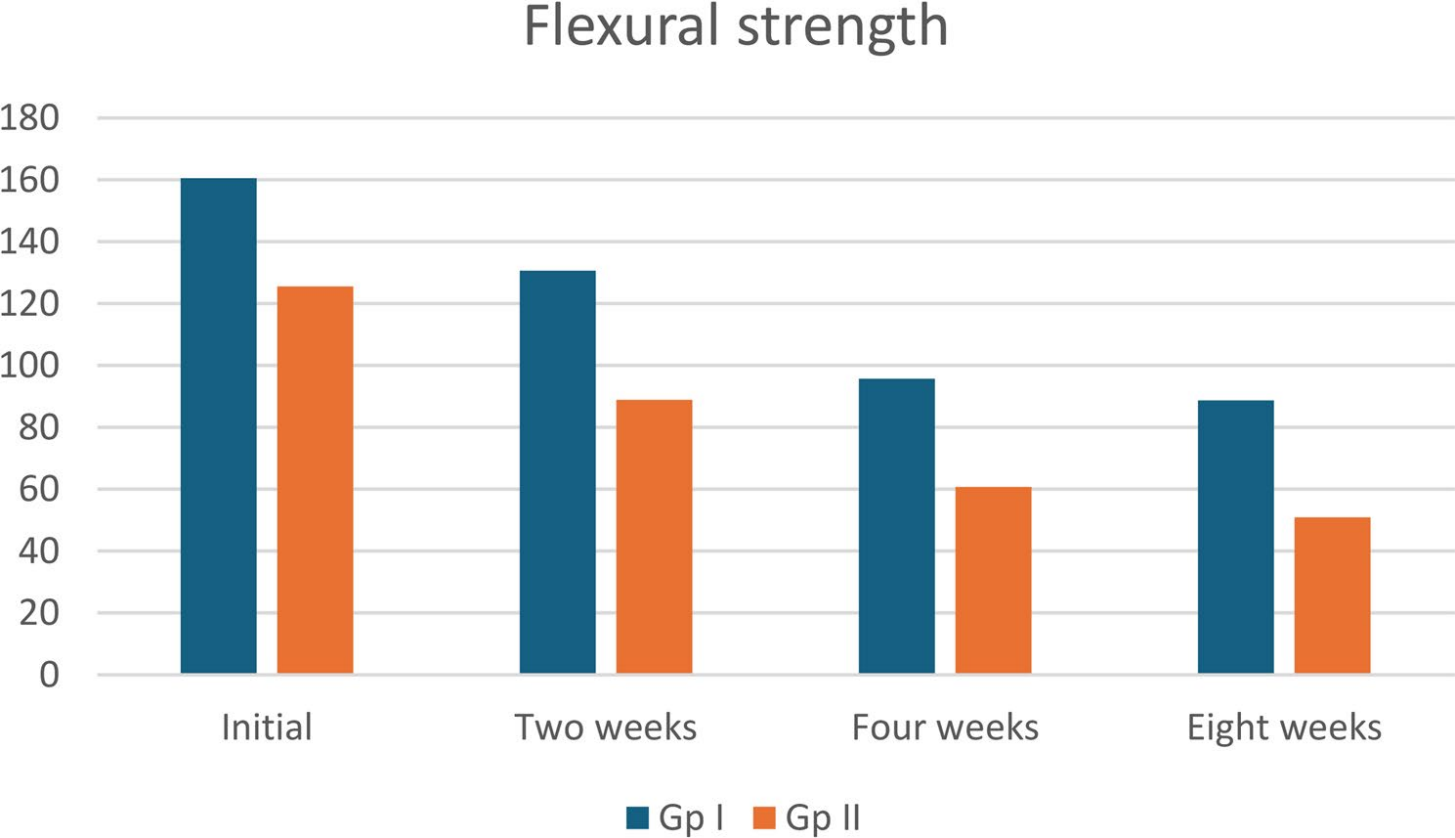
Histogram of the statistical analysis of the flexural strength (Mpa) among studied groups

Group I (Mg1Zn0.6Ca) alloy exhibited the higher initial flexural strength versus group II (160.56±0.03, 125.54±0.03), respectively. It maintained higher strength than group II (Mg6Zn0.6Ca) throughout the 8-week immersion period (160.56, 130.67±0.03, 95.75±0.03, 88.75 Mpa) respectively. Both alloys experienced a significant decline in flexural strength over time, as shown in Table (9) and Fig. (11).

**Table 9:** The statistical analysis of the flexural strength (Mpa) among studied groups

Flexural strength	Gp I(Mg 1zn0.6ca)	Gp IIMg 6zn0.6ca	*p*-value for each row
	Mean ±SD	Mean ±SD	
Initial	160.56±.03^A^	125.54±.03^A^	<0.001
Two weeks	130.67±.03^B^	88.85±.3^B^	<0.001
Four weeks	95.75±.03^C^	60.75±.03^C^	<0.001
Eight weeks	88.75±.03D	50.87±.03^D^	<0.001
*p*-value for each column	<0.001	<0.001	

Friedman test for comparison along the time interval, p-value for flexural strength along the same group; represents the significance along the same column by upper letter. the values with the same letter are non-significant. Whereas the diffierent letter are significant

*SD* standard deviation, *p* probability level

## Discussion

For maxillofacial applications, this study assessed the possibility of biomimetic new bone regeneration that coincided with strength development when the Zn proportion increased in Mg-Zn-Ca alloys (Mg1Zn0.6Ca [Group I] vs. Mg6Zn0.6Ca [Group II]). The clinical value of magnesium alloys is limited by their rapid corrosion, despite their bone-like mechanical characteristics, biodegradability, and biocompatibility for maxillofacial regeneration. Zn enhances corrosion resistance and Ca promotes mineralization in ternary Mg-Zn-Ca systems, which balance degradation with osteogenic activity [29]. 

Biodegradation and potentiodynamic rates revealed Zn-dependent divergence (anodic: Mg → Mg²⁺ + 2e⁻; cathodic: 2 H₂O + 2e⁻ → H₂ + 2OH⁻) caused both alloys to peak at two weeks (Group I: 1.81 ± 0.43 mm/year; Group II: 2.89 ± 0.39 mm/year; *p* < 0.001). XRD demonstrated that Mg (OH)₂ films formation that accompanied by local pH elevation: Group I’s α-Mg declined 83% → 2.95% after 8 weeks, whereas Group II’s 58.8% → 10.9% indicated quicker phase change with greater Zn. Due to the initial i_corr (72.46 vs. 124.67 µA/cm²) prior to passivation, potentiodynamic rates significantly overestimated weight loss (Group I: ≈134–1527%; Group II: ≈147–258%), with weight loss capturing integrated degradation through developing films. (Tables 3 and 4) [26, 30].

Alloys were further characterized by the evolution of their surface topography and structure phase’s distributions. Both types of alloys revealed apparent inconsistencies reflect dynamic corrosion driven roughening and passivation during SBF immersion, unique to each alloy’s content. With a baseline roughness of 15.69 ± 0.03 μm compared to 2.84 ± 0.03 μm (*p* < 0.001) for Group I, Group II’s high Zn content (> 3 wt%) promoted galvanic corrosion through Ca₂Mg₆Zn₃ cathodes and a higher i_corr. Through ZnO/Zn(OH)₂ protection, Group II smoothed rapidly (15.69 → 4.87 μm at 2 weeks), increased moderately (8.05 μm at 4 weeks), and then cracked (25.69 μm at 8 weeks) due to HA delamination. Group I’s moderate Zn refined microstructure, on the other hand, stabilizes Ca/P ratios (0.29 ± 0.03) and promotes bioactivity. It is stable early (2.81 μm), peaks at 33.92 μm (4 weeks; HA porosity), and gradually passivates to 16.64 μm (8 weeks). This very clear as group I potentiodynamic severely overestimates (134–1572%) where passivation occurred with weight loss (1.81→ 0.26 mm/year). Group II potentiodynamic moderately overestimates (147–258%) and limited protection, Weight loss stabilizes ~ 2 mm/year vs. initial 2.89, Table (5). This is consistent with the duality of zinc: high zinc provides temporary protection, whereas low zinc synchronizes long-term HA/strength [31].

FTIR analysis of biomimetic mineralization/matrix formation Figs. (6, 7) revealed similar HA deposition (PO₄³⁻, CO₃²⁻, Mg (OH)₂), but Zn-dependent maturity and affected by SBF type used. The phosphate level remained extremely low (normal range: 0.0025-0.02%; reference: 120.79 ± 0.45; Table 10). Group II remained immature (44%; 37.7% crystallinity), but Group I achieved aberrant maturation (161% CO₃/PO₄ normal at 8 weeks; crystallinity 65.5%) by progressive ordering. According to Li et al. (2009), magnesium dissolution induced alkaline sites (pH > 9), attracting polar organics via physisorption of glucose hydrolysis products (C₆H₁₂O₆ + OH⁻ → CH₃C(O)-/amide I fragments [0.008% normal]; Mg(OH)₂···O = C-NH-CH₃ → HA integration), HA precipitation: Ca²⁺ + PO₄³⁻ then supersaturating → Ca₁₀(PO₄)₆(OH)₂ (apetite-like) with a temporal progression of 2 weeks (porous adsorption, maturity 77.5%); 4 weeks (HA entrapment, mineral/matrix 216.5%); and 8 weeks (buried organics, 151%/17%) Although unstable after 8 weeks, Group I’s globular amorphous calcium phosphate/honeycomb Mg (OH) ₂, Fig. (8).

**Table 10:** The statistical analysis for bone quality parameters of group I and group II after immersion in SBF at different time intervals

Parameter of bone quality		normal reference Cancellous bone	Group IA(Mg1Zn0.6Ca)			Group IIA(Mg6Zn0.6Ca)			*p*-value for each raw
Time intervals			B2w	C4w	D8w	B2w	C4w	D8w	
Mineral Quality	Mineral content (Po_4_)	120.79±0.45	0.003±.0002(0.0025%)	0.025±0.001(0.02%)	0.017±0.000(0.014%)	0.02±0.003 (0.016%)	0.025±0.001(0.02%)	0.017±0.000(0.014%)	<0.001
	HA crystal maturity CO3/PO4	1.54±0.48	1.20±0.40 (77.9%)	0.83±0.26 (53.9%)	2.48±0.85 (161%)	0.38±0.47(24%)	0.31±0.32(20%)	0.67±1.07(44%)	<0.001
	HA crystallinity	24.9±0.18	5.14±0.18(20.6%)	1.33±0.01 (5.3%)	16.32±0.32 (65.5%)	14.31±0.19(57.4%)	14.31±0.19(57.4%)	9.40±0.28(37.7%)	<0.001
Matrix quality	Amide I	10.28±0.001	0.001±0.002(0.008%)	0.001±0.002 (0.008%)	0.001±0.002(0.008%)	0.001±0.000(0.008%)	0.001±0.000(0.008%)	0.001±0.000(0.008%)	<0.001
Mineral /matrix Ratio	PO4^-3^/Amide	11.75±0.002	3.39±0.33(28.9%)	25.44±0.34(216.5%)	17.76±1.58(151.1%)	20.44 ±0.29 (20%)	25.44±0.34 (25%)	17.76±1.58(17%)	<0.001

Degradation was mechanically associated with maxillofacial longevity (cortical loads of 50–150 MPa). Despite micro-galvanic effects at higher Zn Group I maintaine, d flexural strength (88.75 ± 0.03 MPa at 8 weeks) through optimal Zn (1 wt%) grain refinement/solid-solution strengthening (Hall-Petch; 180–220 MPa Mg-1Zn) and HA reinforcement. Group II failed load bearing despite rapidly early protection, degrading to 50.87 ± 0.03 MPa due to brittle Mg₄Zn³/Mg₂Zn₃ (> 15 vol%), it revealed long-term embrittlement [29, 32].

Higher Zn enhanced regeneration strength instead of synchronizing it, which was contrary to the hypothesis. While Group II is only appropriate for short-term orbital contouring (marginal mandibular; suitable orbital; ASTM F382 unsuitable maxillary appliances), Group I’s superior mineral/matrix (28.9-216.5%; peak 216.5% at 4 weeks), HA progression (65.5% crystallinity), and Ra stabilization optimize load-bearing maxillofacial reconstruction. According to Group I, (1 weight% zinc) is ideal for biomimetic bone regeneration with mechanical competence for suitable long- lasting load- bearing maxillofacial appliances [14, 33, 34, 35].

## In Summary

The following Table (11) shows the correlation of each variable property studied and their relevance to bone regeneration (Trade-off Analysis):

**Table 11:** Variables studied and their relevance to bone regeneration (trade-off Analysis)

Property	Mg1Zn0.6Ca (Group I)	Mg6Zn0.6Ca (Group II)	Relevance to Bone Regeneration and Clinical implication
Zn Content (wt.%)	~1	~6	I: Moderate Zn improves corrosion resistance.II: high Zn accelerates corrosion
Microstructure	α-Mg dominant, controlled intermetallics	More intermetallic phases (Ca₂Mg₆Zn₃)	I: Stable phases promote HA formation.II: excessive intermetallics cause galvanic corrosion
Electrochemical Corrosion	Low icorr, high resistance, low rate	High icorr, low resistance, high rate	Matches phase evolution, bone quality, and FTIR findings
Corrosion and Degradation Rate	1- ~4.23(mm/yr)2- Slow, self-limiting (Rapid drop, stabilizes at low value).	1- ~7.37 (mm/yr)2-Rapid, uncontrolled(High, decreases slightly, remains high).	I: Ensures mechanical support during healing (Long term)II: transient.
XRD/FTIR/Bone Quality	HA and Mg (OH)₂ form, improved mineral	Mg (OH)₂ dominates, no HA, poor mineral	I: Phase evolution dictates corrosion and bone healing
HA Formation rate	Gradual, mature crystallinity	Minimal, unstable phases	I: Enhances osteointegration II: limited bone contact
Ca/P Ratio	Progressive (0.29 → 0.68)	Declining (1.42 → 0.35)	I: Supports bone-like mineral deposition
Surface Mineralization	Progressive HA and Ca-P formation	Unstable mineral layers	I: Stable mineralization enhances osteointegrationII: transient
Surface Roughness	Moderate, beneficial changes	Erratic, disruptive	I: Facilitates cell adhesion and growthII: transient
Flexural Strength	Higher retention (88.75 MPa at 8w)	Rapid loss (50.87 MPa at 8w)	I: Maintains load-bearing capacity.II: Short-term

## Conclusions

Under the limitations of this study, Mg1Zn0.6Ca (Group I) demonstrated superior biomimetic synchronization for maxillofacial bone regeneration, achieving controlled long-term degradation (weight loss corrosion rate reduced from 1.81 to 0.26 mm/year over 8 weeks), mature HA formation (65.5% crystallinity, 161% crystal maturity by 8 weeks), and preserved mechanical competence (88.75 ± 0.03 MPa flexural strength), demonstrating its suitability for non-load-bearing orbital floor repair (30–70 MPa) and load-bearing applications such as maxillary buttress fixation (80–120 MPa) and mandibular angle reconstruction (100–150 MPa).

On the other hand, Mg6Zn0.6Ca (Group II) showed rapid initial passivation (Ra ∼4.87 μm at 2 weeks) but unsustainable performance because of premature embrittlement (50.87 ± 0.03 MPa), immature HA (44% maturity, 37.7% crystallinity decline), and persistent high degradation (~ 2 mm/year), which limited its use to short-term, non-load-bearing contour restoration (orbital floor; 4–6 week resorption profile). Instead of improving the regeneration-strength coupling as expected more Zn (6 wt%) disturbed it, demonstrating that 1 wt% Zn is the ideal amount for biomimetic maxillofacial implants in accordance with ISO 10993-5 biomechanical thresholds.

## Recommendation

Group I is best suited for orthopedic implants that require bioactive surfaces and long-term mechanical stability, whereas Group II might require alloy refining to mitigate rapid corrosion.

## Data Availability

The authors announce that the data supporting the results of this study exist within the article.

## Abbreviations

SBF: Simulating body fluid
XRD: X-ray diffraction analysis
SEM: Scanning electron microscope
EDX: Energy dispersive x-ray analysis
(CMRDI): Central metallurgical research and development institute
HA: Hydroxyapatite
